# Double Valve Infective Endocarditis Complicated by Systemic Arterial Embolization

**DOI:** 10.7759/cureus.19119

**Published:** 2021-10-29

**Authors:** Genesis Perez Del Nogal, Bibek Bakhati, Joshua A Ronen, Alejandra Garcia Fernandez

**Affiliations:** 1 Internal Medicine, Texas Tech University Health Sciences Center, Odessa, USA; 2 Internal Medicine, University of California San Francisco School of Medicine, San Francisco, USA; 3 Division of Hospital Medicine, University of California San Francisco Medical Center, San Francisco, USA; 4 Critical Care, Texas Tech University Health Sciences Center, Odessa, USA

**Keywords:** valvular replacement, endocarditis, infective endocarditis, systemic emboli, double valve infective endocarditis, staphylococcus aureus endocarditis

## Abstract

A 26-year-old male with a past medical history of intravenous opioid abuse was admitted with the diagnosis of double valve infective endocarditis and methicillin-resistant *Staphylococcus aureus* bacteremia. Imaging, excluding the head, was indicative of systemic arterial embolization, as abscesses had developed in the retroperitoneum and prostate. There was evidence of splenic infarct, and the presence of extensive pulmonary infiltrates indicative of showering septic foci from the heart. Antibiotic therapy was started and a transesophageal echocardiogram demonstrated mitral and tricuspid valve vegetations with a preserved ejection fraction. Fortunately, the valvular repair was successful and artificial valves were not needed. The patient had an uncomplicated postoperative course in the intensive care unit and was transferred back to the ward in stable condition. He remained on the ward for six weeks due to his unfunded status until his antibiotic course and physical rehabilitation were completed.

## Introduction

Infective endocarditis (IE) is an uncommon infection of the heart valves (native or prosthetic) or endocardium that carries high morbidity and mortality. Incidence in the United States has been increasing for the past decade [1,2]. Risk factors include male sex, intravenous drug use (IVDU), poor dentition or infection as well as patients with structural heart disease, prosthetic valves or intracardiac devices, history of IE, and chronic hemodialysis [3]. Per Moreillon and Que, IVDU is associated with approximately 90% of cases of right-sided IE (RSIE) and approximately 20% of cases of left-sided IE (LSIE) [4]. *Staphylococcus aureus* is the most common cause of endocarditis, and metastatic infections are described in the brain, eye, spleen, kidney, spine, and bony architecture [5].

## Case presentation

A 26-year-old male with a past medical history of methicillin-resistant *S. aureus* (MRSA) cellulitis in childhood secondary to leg trauma, chronic tobacco habituation of five pack-years, and IV drug use was transferred from an outside facility for a higher level of care. The patient was being treated with vancomycin for bilateral community-acquired pneumonia, MRSA bacteremia, and left forearm cellulitis. Initial blood cultures from the sending facility were positive for MRSA. Transthoracic echocardiogram (TTE) at the outside facility showed echogenic densities on the mitral and tricuspid valves suggestive of infective endocarditis. On admission to the receiving facility, the patient endorsed generalized weakness and myalgias, left shoulder pain, subjective fevers, and a productive cough for one week. 

On physical examination, the patient was hemodynamically stable, no oxygen requirements, awake, alert, oriented, in acute distress due to generalized body pain. Cardiothoracic examination revealed coarse breath sounds bilaterally at lung bases, and S1/S2 heart sounds were heard without murmur, gallop, or rub. Furthermore, he was in a regular rate and rhythm. His abdomen was soft, non-distended, yet diffusely tender to palpation, with no guarding or rebound. Skin examination showed Osler's nodes in the left fingertips, Janeway lesions on both feet, and bilateral forearms with track markings.

Admitting diagnosis to the general medicine wards was MRSA bacteremia secondary to infective endocarditis of the mitral and tricuspid valve in the setting of the known history of IVDU. Cardiology, cardiothoracic surgery, infectious disease, and orthopedic surgery consultations were sought.

**Figure 1:**
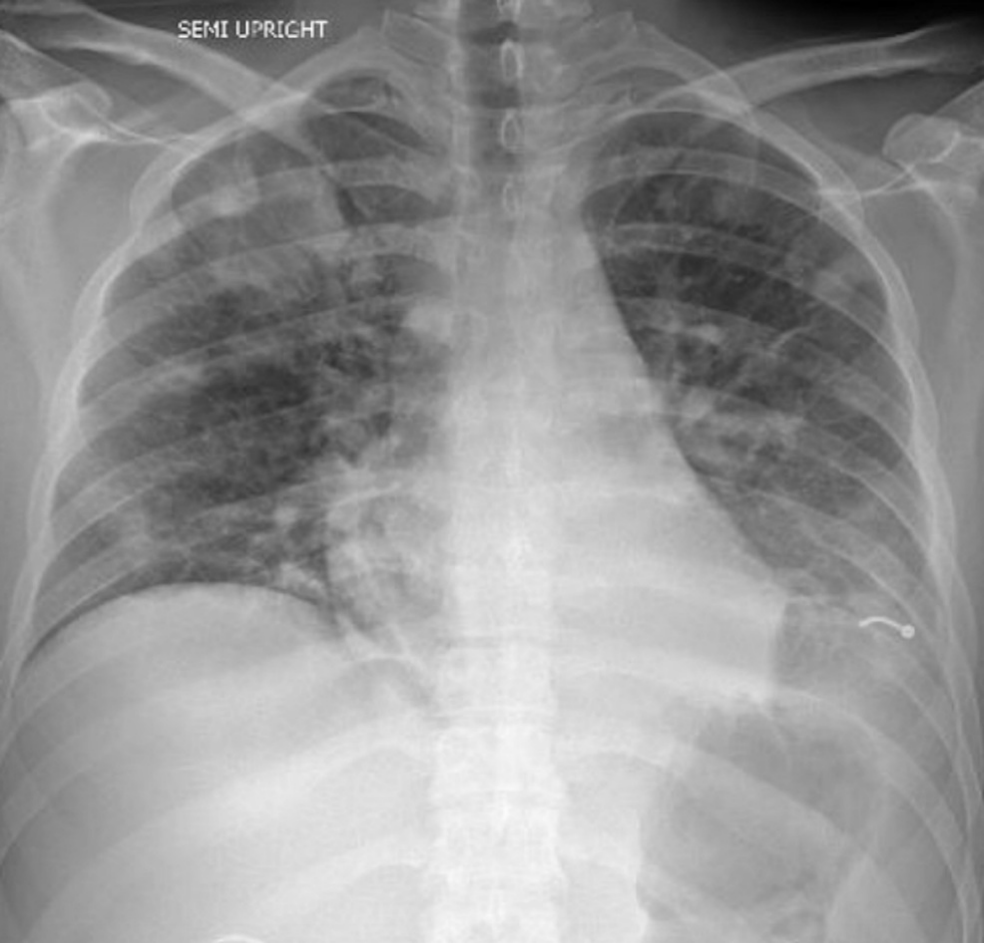
Bilateral diffuse nodular infiltrates on chest x-ray. Artifact noted at the left pulmonary base.

**Figure 2:**
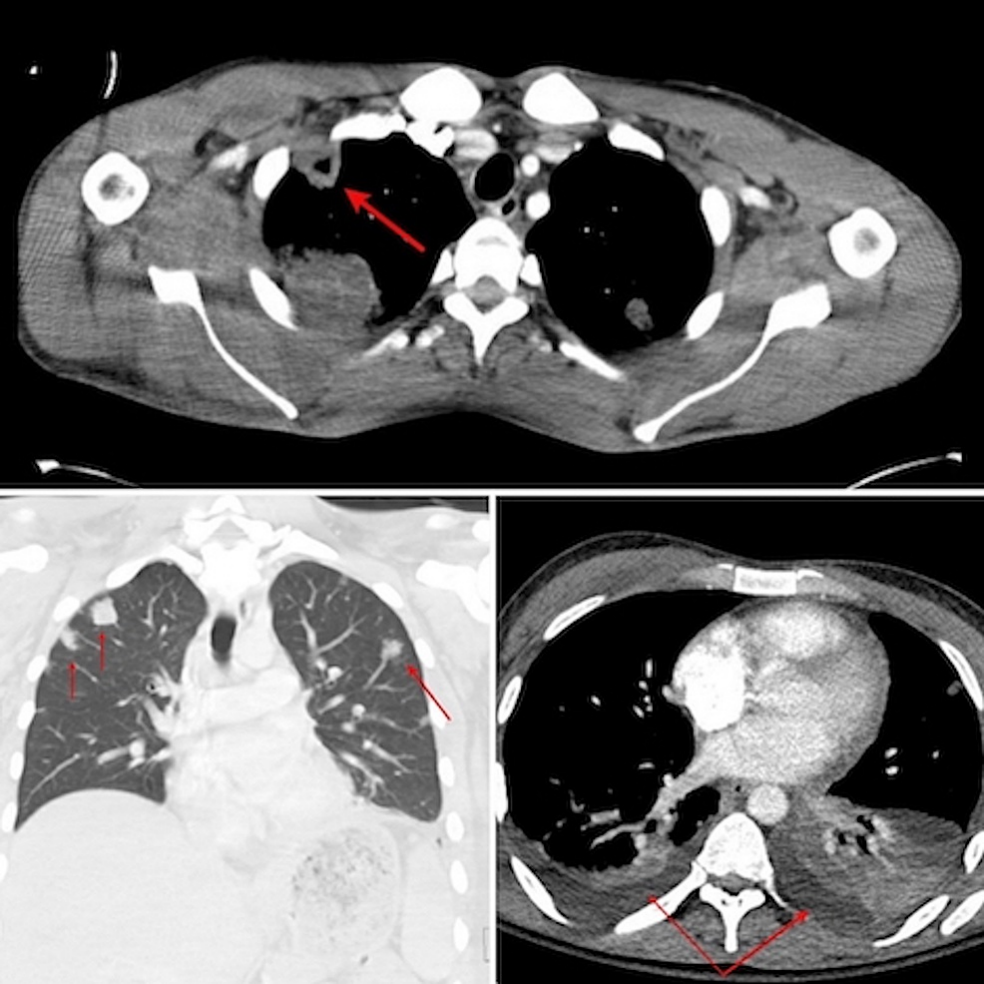
CT scan of the chest (red arrow) shows a cavitary lesion on top, bilateral septic (red arrow) pulmonary emboli (left side of the image), and bilateral pleural effusions (red arrow) more prominent on the left than the right side (right side of the image). CT: Computed tomography.

Chest x-ray (CXR) on admission revealed bilateral nodular infiltrates (Figure 1). Computed tomography (CT) scan of the chest demonstrated bilateral multilobar pulmonary lesions representing septic pulmonary emboli and a cavitary defect, accompanied by bilateral pleural effusions more prominent on the left than the right side (Figure 2).

**Figure 3:**
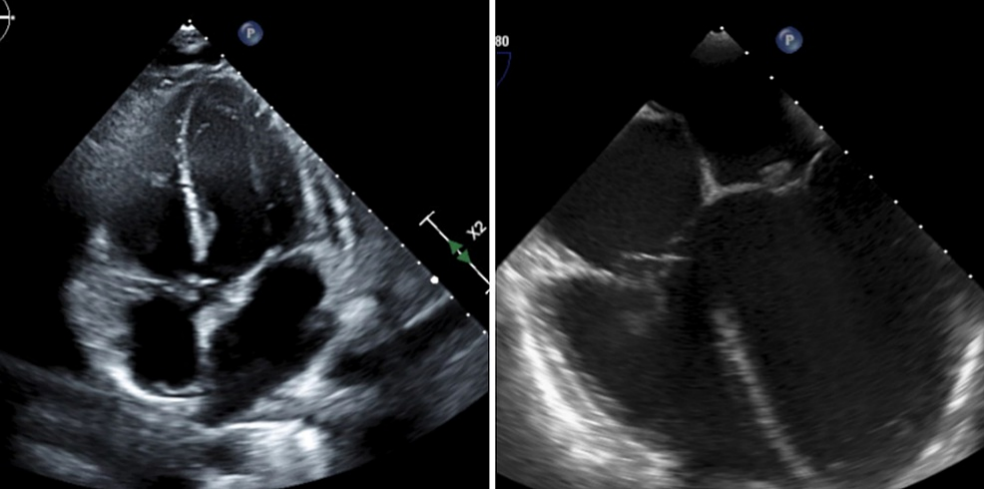
TTE and TEE showing mitral and tricuspid valve vegetations. The left-sided image showed a transthoracic echocardiogram (TTE) and the right-sided image a transesophageal echocardiogram (TEE).

A transesophageal echocardiogram (TEE) was done and yielded a normal left ventricular ejection fraction (LVEF) of 60 to 65%. However, it revealed vegetations on the mitral valve (1.4 cm × 1.3 cm on the atrial aspect of the tip of the posterior leaflet) and tricuspid valve (1.3 cm × 2.3 cm on the right ventricular aspect of the base of the posterior leaflet) (Figure 3).

**Figure 4:**
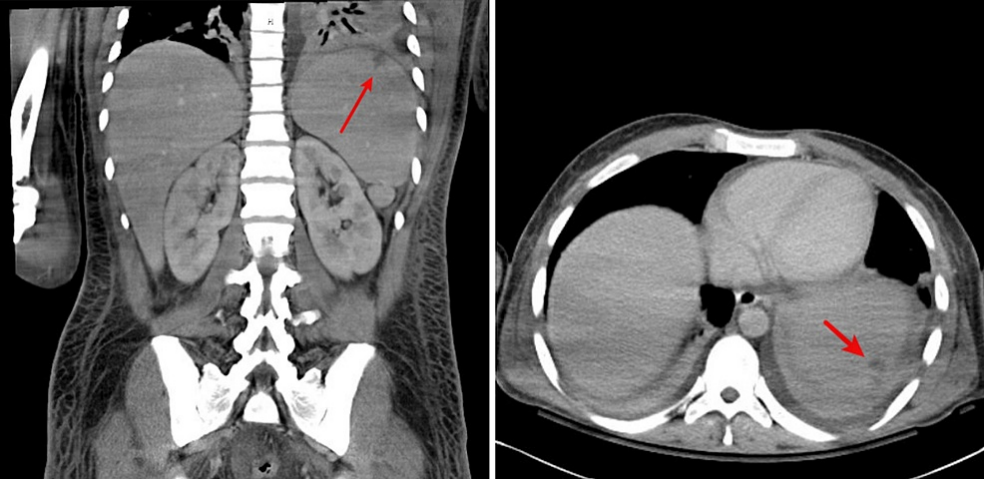
CT scan of the abdomen exhibits a small wedge-shaped (indicated by the arrow) peripheral splenic infarct. CT: computed tomography.

**Figure 5:**
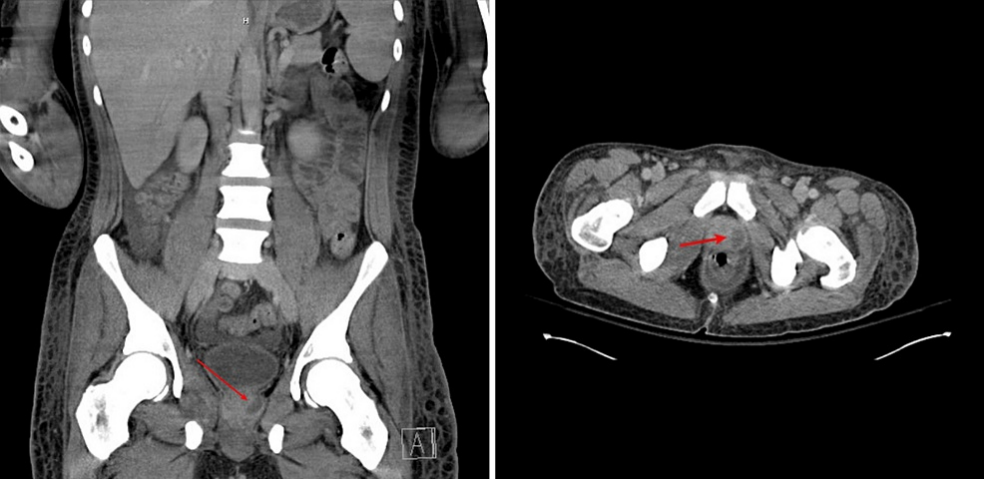
CT scan of the abdomen evidence (indicated by the arrow) left prostate abscess. CT: computed tomography.

Over his hospital course pre-operatively, the patient complained of generalized pain. CT abdomen exhibited diffuse hepatomegaly, mild splenomegaly, and a small wedge-shaped peripheral splenic infarct (Figure 4). In addition, findings were consistent with emboli to small vessels of the bilateral renal upper poles and the left lateral aspect of the prostate gland suspicious for a small abscess (Figure 5). Lastly, enlargement and low-density heterogeneity of the right internal obturator muscle represented a small intramuscular abscess. A magnetic resonance imaging (MRI) of the full spine with and without gadolinium contrast was negative for any evidence of epidural spinal cord compression. While daily skin and joint exams ensued, arthrocentesis of his left shoulder or other joints to rule out further metastasis of infection was not needed according to orthopedic surgery.

This patient’s case of IE complicated with systemic arterial embolization to lungs, spleen, kidneys, extremities, prostate, bladder, and left internal obturator was treated with vancomycin and piperacillin-tazobactam initially. As hematogenous dissemination of MRSA was also detected by urine culture pre-operatively, he was switched to daptomycin for the remaining duration of his antibiotic course, and piperacillin-tazobactam was discontinued. Total antibiotic treatment duration was determined to be eight weeks from his last negative blood culture. Due to evidence of valvulopathy while not clearly evident on physical exam, the valvular replacement was considered as one of the treatment options. The patient was deemed high risk due to the presence of disseminated disease. Nonetheless, the patient underwent excision of mitral valve left leaflet vegetations, repair of mitral valve perforation, excision of the tricuspid valve chordal vegetation, and repair of tricuspid valve chordal rupture. Prosthetic valve implantation was not needed, sparing him from postoperative therapeutic dose anticoagulation. His perioperative course was uncomplicated and he was then transferred to the intensive care unit (ICU). Routine hospital-based postoperative cardiac surgery ensued. 

After clearance by the intensivist, he was transferred back to the general medical ward. The patient remained stable, however, due to socioeconomic challenges and lack of stable housing options locally, he remained hospitalized for six weeks until completion of antibiotic therapy and was discharged without further complaints.

## Discussion

Infective endocarditis (IE) is an uncommon infection of the cardiac valves (native or prosthetic), endocardium, or cardiac implantable electronic devices (CIED), including pacemakers and implantable cardioverter-defibrillators (ICDs) [6,7]. It can be right-sided, left-sided, or device-related based on the localization of infection and depending on the nature of the valve involved. Another way to categorize IE is according to the mode of acquisition-community-acquired, nosocomial, or intravenous drug use (IVDU) associated. The previous classification of IE relative to the onset of disease (acute, subacute, chronic) is no longer preferred as it provides little benefit to diagnostic workup and empiric treatment [6,8,9].

The pathognomonic feature of IE is vegetation, which is composed of platelet-fibrin deposition and bacterial colonies in the affected valvular endothelium, endocardium, or cardiac devices. Vegetations can lead to valvular destruction, embolization, metastatic infection, and varied immunologic manifestations [7]. More expeditious indications for surgery include decompensated heart failure, perivalvular abscesses, persistent fever greater than one week (>101.4 degrees Fahrenheit), and/or presence of multi-drug resistant or fungal organisms.

The incidence of this rare entity is about three to seven cases per 100,000 population yearly [8,10]. Rheumatic heart disease continues to be the leading etiological factor for the development of IE in developing countries and it mainly affects the younger population. On the other hand, due to better living conditions and the availability of antibiotics against penicillin-sensitive streptococcal pharyngitis, rheumatic heart disease has become an uncommon risk factor in developed countries. Intravenous drug use, degenerative valve disease, and congenital heart disease are major contributing factors in developed nations and the affected population tends to be older [6-8].

Right-sided native valve infection is less common and comprises roughly 10% of overall IE cases. Concomitant left- and right-sided endocarditis, as seen in the case described here, are found in approximately 13% of cases [11]. IVDU accounts for almost 90% of cases of right-sided IE and 20% of left-sided IE [4].

Around 80-90% of the endocarditis cases are due to gram-positive cocci belonging to *Staphylococcus*, *Streptococcus*, and *Enterococcus* species [6]. *S. aureus*, especially methicillin-resistant, is a common cause of IVDU associated IE, particularly if the tricuspid valve is involved. In addition to these micro-organisms, LSIE in drug addicts can be due to *Pseudomonas aeruginosa*, *Candida* sp. and infrequently caused by *Bacillus*, *Lactobacillus*, and *Corynebacterium* sp. Polymicrobial infection is also prevalent in this population [8].

The most common clinical findings of IE are fever (in about 90%) and cardiac murmur (in approximately 75-85% of cases) [6,8]. The classic immunologic and microembolic findings such as Janeway lesions, Roth spots, Osler nodes, and splinter hemorrhages are rare and found in around 5-10% of cases [6-8]. Hematogenous seeding can involve the musculoskeletal system, meninges, skin, spleen, and kidneys. Embolic phenomenon leading to end-organ ischemia can be clinically evident in approximately 50% of cases and are seen more frequently with *S. aureus* associated IE, >10 mm and mobile vegetations and endocarditis involving the anterior leaflet of the mitral valve. Tricuspid valve endocarditis often manifests with fever and septic pulmonary emboli, but cardiac murmur and peripheral findings are typically absent [9]. Our patient had an extensive history of IVDU and presented with MRSA bacteremia and findings associated with systemic arterial embolization due to hematogenous seeding of infection.

Integration of clinical, microbiological, and echocardiographic features is required to diagnose IE. The modified Duke criteria, consisting of major and minor components, is an invaluable tool that incorporates all these features and aids in the diagnosis [6-8]. However, clinical judgment still supersedes this tool, especially in cases of prosthetic valve endocarditis (PVE), CIED, and right-sided IE [6,8]. Blood cultures are an important component of major criteria that not only carry diagnostic value but also guide appropriate antibiotic therapy [6-8]. Prior to the administration of empiric antibiotics, three sets of blood cultures drawn from different venipuncture sites are recommended [8]. Another component of major criteria is detecting new valvular regurgitation or demonstration of vegetation, abscess, or new partial dehiscence of present prosthetic valves via TTE [6-8]. TTE is a noninvasive and useful initial tool. Transesophageal echocardiogram (TEE) is beneficial in indeterminate cases and is also highly sensitive and specific for detecting intracardiac complications and diagnosing PVE and CIED endocarditis [6,8]. Minor criteria comprise documentation of fever, predisposing conditions, vascular phenomena, immunologic manifestations, and microbiologic evidence of organisms that do not meet major criteria. A combination of two major, one major and three minor, or five minor criteria can help us diagnose IE. Alternatively, a definitive diagnosis can be made with microbiological and histological analysis of pathologic specimens [6-8]. The patient in the case described fulfilled two major criteria, he had positive blood cultures and vegetations that were evidenced on the TEE.

IE requires multidisciplinary input for treatment, mainly with the help of infectious disease specialists, cardiologists, and cardiothoracic surgeons [6]. Parenteral antibiotics are the cornerstone of therapy and should be continued for six to eight weeks from the last negative blood cultures [6,8]. Surgical treatment is mainly indicated for the prevention of systemic embolism, valvular dysfunction causing heart failure, and uncontrolled infection [6,7]. Early surgery has better survival benefits with life-threatening indications [8]. Consequently, this confirmed that early treatment improves the prognosis of patients with double valve infective endocarditis, and valvular repair with prolonged antibiotic therapy is a suitable option for the treatment of this pathology.

## Conclusions

Right-sided IE is more common in IV drug users. Left-sided IE, although uncommon, should be suspected in these patients, especially if they present with symptoms of systemic emboli.

TEE should follow TTE if it is suboptimal and there is a strong suspicion of multivalvular involvement (in the setting of bacteremia), which was otherwise not visualized in TTE. 

Multivalvular involvement, more specifically, both right and left-sided valve involvement, is extremely rare and can be life-threatening if treatment is delayed. Early surgical intervention along IV antibiotic therapy is recommended in this cohort of patients to improve morbidity and mortality.
